# Bacterial composition of midgut and entire body of laboratory colonies of *Aedes aegypti* and *Aedes albopictus* from Southern China

**DOI:** 10.1186/s13071-021-05050-4

**Published:** 2021-11-27

**Authors:** Datao Lin, Xiaoying Zheng, Benjamin Sanogo, Tao Ding, Xi Sun, Zhongdao Wu

**Affiliations:** 1 Department of Parasitology, Zhongshan School of Medicine, Sun Yat-Sen University, Guangzhou, Guangdong China; 2 Key Laboratory of Tropical Disease Control, Ministry of Education, Provincial Engineering Technology Research Center for Diseases-Vectors Control, Guangzhou, Guangdong China; 3 Chinese Atomic Energy Agency Center of Excellence on Nuclear Technology Applications for Insect Control, Sun Yat-sen University, Guangzhou, China

**Keywords:** Mosquito, Vector, High-throughput sequencing, Microbiome, Midgut, Entire body

## Abstract

**Background:**

*Aedes aegypti* and *Aedes albopictus* are invasive mosquito species and significantly impact human health in southern China. Microbiota are confirmed to affect the development and immunity of mosquitoes. However, scientists have focused more on midgut microbiota of female mosquitoes and bacterial differences between female and male *Aedes* mosquitoes. The relationship between the midgut and entire body microbiota of *Aedes* is unclear. In this study, we collected mosquito samples reared under the same laboratory conditions and compared the microbial composition of midgut and entire bodies of *Aedes aegypti* and *Aedes albopictus* using 16S rRNA gene sequencing.

**Methods:**

In this study, we collected mosquito samples reared under the same laboratory conditions and compared the microbial composition of midgut and entire bodies of Aedes aegypti and Aedes albopictus using 16S rRNA gene sequencing.

**Results:**

A total of 341 OTUs were identified, showing that *Proteobacteria* was the dominant phylum and *Methylobacterium* the dominant genus in both *Aedes aegypti* and *Aedes albopictus*. The bacterial diversity and community structures of the entire bodies were similar between males and females in both *Aedes aegypti* and *Aedes albopictus*. Conversely, the bacterial compositions of male and female *Aedes aegypti* and *Aedes albopictus* were significantly different. NMDS analysis, UPGMA analysis, diversity indices and OTU distribution demonstrated that compositions and structures in midgut microbiota were similar but significantly different in the entire bodies of *Aedes aegypti* and *Aedes albopictus*. Functional prediction analysis showed that metabolism and environmental information processing were the dominant KEGG pathways at level 1. Our study showed that there were significantly different level 2 and 3 KEGG pathways in the midgut microbiota (16 level 2 and 24 level 3) and the entire bodies (33 level 2 and 248 level 3) between female *Aedes albopictus* and *Aedes Aegypti*.

**Conclusions:**

Our findings that *Aedes aegypti* and *Aedes albopictus* reared in the same laboratory harbor a similar gut bacterial microbiome but different entire body microbiota imply that the gut microbiota of adult mosquitoes is environmentally determined regardless of the host genotype, but the entire body microbiota is more genetically determined. Our findings improved the understanding of the microbiota in the entire and partial tissues of *Aedes* mosquitoes.

**Graphical abstract:**

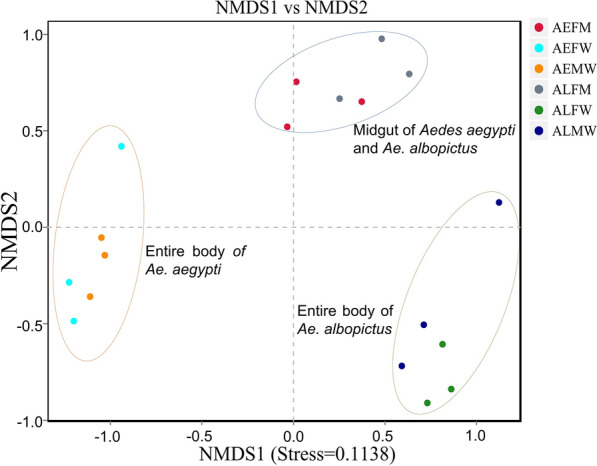

**Supplementary Information:**

The online version contains supplementary material available at 10.1186/s13071-021-05050-4.

## Introduction

*Aedes aegypti* and *Ae. albopictus*, well-known invasive mosquito species, have rapidly expanded and spread to most countries globally in the past 40 years [1–3]. These two mosquitoes are important vectors transmitting dengue virus (DENV) [4], Zika virus (ZIKV) [5], West Nile virus (WNV) [6] and chikungunya virus (CHIKV) [7], which cause enormous damage and public health problems to human and animal organisms [1, 8–11].

Recent studies on the microorganisms of mosquitoes have indicated that the midgut harbors a diverse microbiota, which can significantly affect the reproduction, development, digestion, nutrition and metabolism, immunity, behavior and other physiological functions of their hosts [12–14]. Previous studies have reported that the composition of midgut-derived bacteria is related to the host and parasite interaction, and the midgut microbiota could block the transmission of pathogens such as Zika virus and *Plasmodium* [5, 15–17]. To investigate the microbial community structure, researchers focused on field-captured and laboratory mosquitoes. They found that the gut bacterial community structures in the adult stage of the field and laboratory mosquitoes were similar [18]. The study revealed that most bacterial genera in wild *Culex pipiens* and laboratory-reared adult *Aedes japonicus* could also be found in other mosquito species, such as *Ae. aegypti* and *Ae. albopictus* [19], indicating that different mosquitoes may share the same microorganisms. Studies on *Ae. aegypti* and *Anopheles gambiae* (*An. gambiae*) revealed that the development of these mosquitoes depended on gut microbiota [20, 21]. These studies focused on the midgut microbiota and the biological aspects of the hosts.

Previous studies have shown that the entire bodies of insects harbor diverse microbiota [22, 23]. These microbiota can influence insect host vector competence [24, 25] and modulate arbovirus transmission in mosquitoes [24, 25]. However, few studies have focused on the entire body microbiota of *Aedes*, and the relationship and difference between the entire and midgut microbiota of *Aedes* mosquitoes are unclear.

To investigate the diversity, composition and network analysis of microbiota in *Aedes* mosquitoes from South China, we used high-throughput sequencing of the 16S rRNA gene. We revealed the bacterial communities and their associations in the entire bodies and midguts of male and female *Aedes* mosquitoes reared under laboratory conditions and investigated the relationship between *Wolbachia* and other microbes of *Ae. albopictus*.

## Methods

### Samples collection

*Aedes* mosquitoes were reared in laboratory conditions. The food consisted of a mixture of lactalbumin hydrolysate, finely ground rat chow and Brewer's yeast (1:1:1). *Aedes* mosquitoes were maintained at 27 °C, RH 80%, with a photoperiod of 14:10 h (L:D) on 10% sucrose solution ad libitum. For this study, we collected samples of the midgut (from females) and entire body (from both males and females) from adult mosquitoes. Before dissection, the entire mosquito was washed three times with sterile distilled water and surface sterilized using 75% ethanol for 1 min [26, 27]. Each sample consisted of five midguts or one entire mosquito as a pool and then was stored in 95% ethanol at − 80 ˚C until further research.

### DNA extraction

Samples collected in a tube were mechanically homogenized using a sterile pestle (an electric homogenizer) in liquid nitrogen. For bacteria community sequencing, total DNA was isolated under a sterile environment according to the protocol of the Hipure Bacterial DNA Kit (Magen, China). Briefly, each sample was put in an Eppendorf tube with 1-mm-diameter inox beads (Qiagen, Germany) and individually crushed using an organization disruptor (Gene Co., Ltd., China). They were removed, and 1 ml extraction STE buffer, 10 μl SDS buffer and 10 μl Proteinase K were added. The samples were heated at 55 ℃ for 1 h. To wipe liquids and proteins, we added 200 μl AL buffer into the tube and then heated it at 70 ℃ for 10 min. Total DNA was precipitated with 250 μl cold absolute ethyl alcohol and centrifugation at 100*g* for 15 s. After these steps, total nucleic acid was transferred into Hipure DNA mini Column I and washed by GW1 and GW1 buffer step by step. Finally, total DNA was resuspended in 30 μl AE buffer. The DNA quality and quantity examinations were conducted using a Nanodrop (Thermo Scientific, USA).

### PCR amplification and sequencing

The 16S rRNA genes of their V3-V4 regions were amplified and sequenced on an Illumina HiSeq 2500 platform. The following primer set was used: forward primer 5'-ACTCCTACGGGAGGCAGCA-3'; reverse primer 5'- GGACTACHVGGGTWTCTAAT-3'. PCR amplification was performed using PrimeStar DNA polymerase (Takara, China). The following PCR cycling conditions were used: denaturation at 95 °C for 5 min, 25 cycles of 95 °C for 30 s, 50 °C for 30 s, 72 °C for 40 s and final extension at 72 °C for 5 min. The PCR products were analyzed by agarose gel electrophoresis. Finally, 16S rRNA gene sequencing was constructed using Biomarker Technologies (Beijing, China).

### Analysis of sequencing data

After the base calling analysis, the original data files from the sequencing platform were transformed into the original sequenced Reads Stored in FASTQ format. QIIME (version 1.8.0) was used to cluster reads into operational taxonomic units (OTUs) and identified at 97% or more similarity [28]. To analyze the alpha diversity, we rarified the OTU table and calculated the species abundance based on two metrics: Ace and Shannon [29]. The unweighted pair group method with arithmetic mean analysis (UPGMA) was performed based on the unifrac distance using QIIME (version 1.9.1). As a measure of beta diversity and similarity, the nonmetric multidimensional scaling (NMDS) graph with Bray-Curtis diversity was done in R with the vegan package [30]. Venn diagram analysis was conducted in R statistical software (version 3.0.3) using the vegan packages. The metabolic functions of the bacterial community were inferred by phylogenetic investigation of communities by reconstruction of unobserved states (PICRUSt) [31]. The correlation network analysis was conducted using the ClusterMaker app in Cytoscape [32]. For range adjustment, all pairwise comparisons between two groups were tested using Student’s *t*-test. *P < 0.05 was considered statistically significant.

## Results

### Mosquito colonies study

The *Ae. aegypti* and *Ae. albopictus* mosquitoes were captured in Hainan and Guangdong provinces, South China (Additional file 1: Fig. S1). The *Ae. albopictus* strain was established from 2000 mosquito larvae collected from 20 different districts of the Guangzhou metropolitan area (about 100 larvae collected per district). Then, they were transferred to a laboratory using a special facility for further study. The field-captured mosquitoes were identified under microscope. The *Ae. aegypti* strain was collected by Guangdong Provincial Center for Disease Control and Prevention, and they offered us this strain. We fed the *Aedes* under laboratory environmental conditions. After rearing > 30 generations, we collected six groups of *Aedes* colonies (Additional file 2: Table S1) to investigate the bacterial composition and diversity shaping the environmental factors in 2017.

### Composition of microbiota in *Ae. aegypti* and *Ae. albopictus*

To analyze the composition and diversity of bacterial communities in both *Ae. aegypti* and *Ae. albopictus* from South China, we performed 16S rRNA gene sequencing of their V3-V4 hypervariable regions. In total, 2,686,929 clean tags were retained and classified into operational taxonomic units (OTUs) at a 97% similarity level after rarefaction and quality filtering. An average of 149,274 clean tags were obtained from sequenced samples. The tags were clustered into 341 OTUs representing 14 phyla, 23 classes, 42 orders, 71 families and 123 genera.

**Fig. 1:**
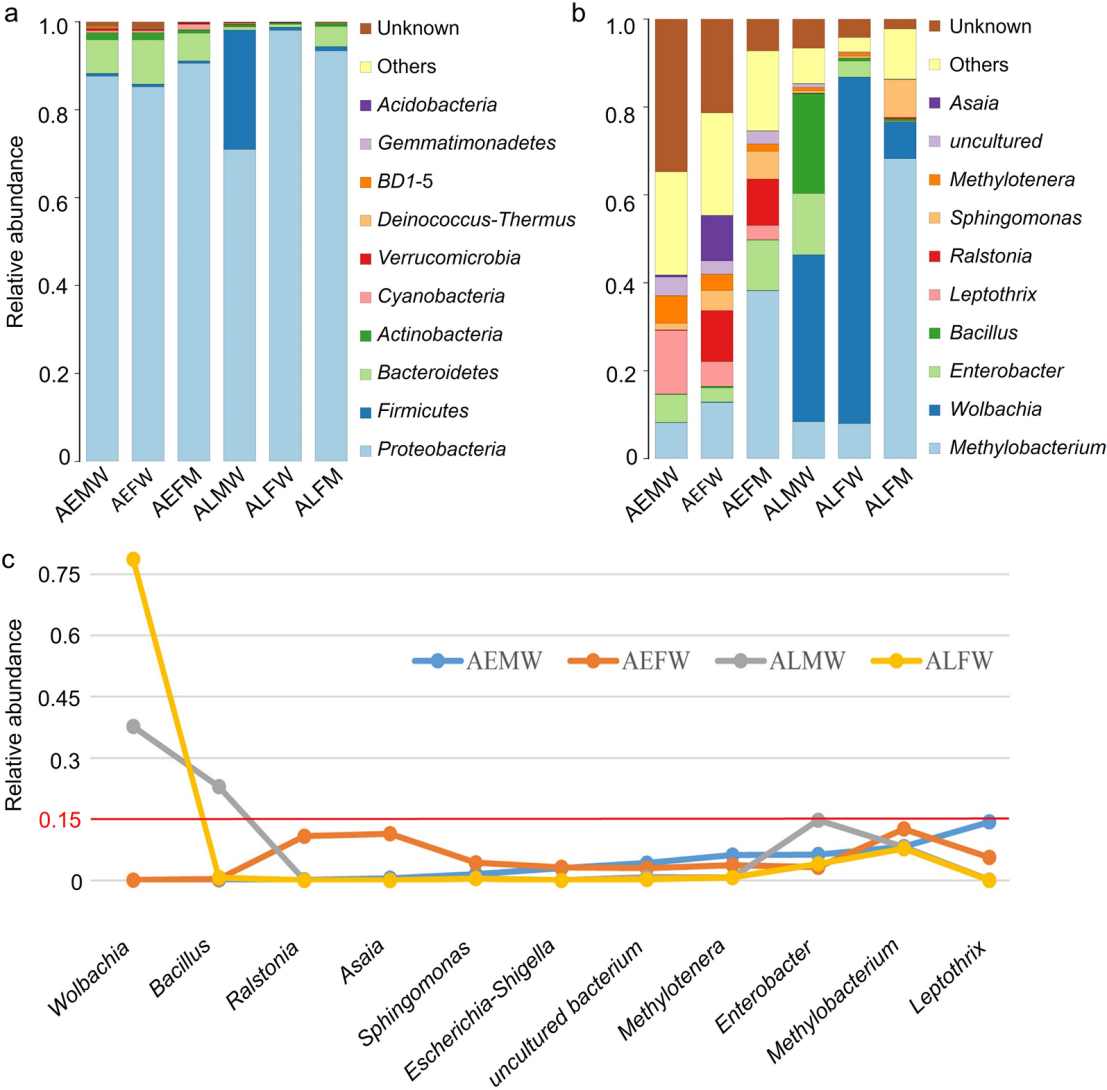
Relative abundance of bacteria at phylum and genus level in *Ae. aegypti* and *Ae. albopictus*, including males and females. **a** Phylum, **b** genus. **c** Average distribution of top ten genera in *Aedes*. Only the ten most common bacterial phyla and genera are shown. AEMW: entire body of male *Ae. aegypti*. AEFW: entire body of female *Ae. aegypti*. AEFM: midgut of female *Ae. aegypti*. ALMW: entire body of male *Ae. albopictus*. ALFW: entire body of female *Ae. albopictus*. ALFM: midgut of female *Ae. albopictus*

We analyzed the differences in the microbial community from both the entire bodies and midguts of *Ae. aegypti* and *Ae. albopictus*. Our findings revealed the relative abundances of the predominant taxa in the six groups (Fig. 1). At the phylum level, the *Ae. aegypti* groups, including the entire bodies of male *Ae. Aegypti* (AEMW), entire bodies of female *Ae. aegypti* (AEFW) and midguts of female *Ae. aegypti* (AEFM), showed close abundances at the main phyla, which were dominated by *Proteobacteria* (87.6%, 85.2% and 90.6%, respectively) and *Bacteroidetes* (7.7%, 10.1% and 6.3%, respectively) (Fig. 1a). The total relative abundances of the other phyla were < 5% in each group. The bacterial compositions of the *Ae. albopictus* groups, including the entire bodies of the male *Ae. albopictus* (ALMW), entire bodies of the female *Ae. albopictus* (ALFW) and the midguts of the female *Ae. albopictus* (ALFM), were dominated by *Proteobacteria* (71.0%, 98.0% and 93.4%, respectively) (Fig. 1b). The ALFM group showed a higher abundance of *Bacteroidetes* (4.6%), and the ALMW population harbored the highest relative abundance of *Firmicutes* (27.2%) compared to those of the other groups. Most microbes in ALFW mosquitoes were identified as the phylum *Proteobacteria*, the relative abundance of which was higher than those of other groups.

At the genus level, the top ten genera in the AEMW group were *Leptothrix* (14.3%), *Methylobacterium* (8.3%), *Enterobacter* (6.3%), *Methylotenera* (6.2%), *uncultured bacteria* (4.2%), *Escherichia*-*Shigella* (3.0%) and *Sphingomonas* (1.5%). The AEMW group harbored diverse microbiota, including *Methylobacterium* (12.6%), *Asaia* (11.4%), *Ralstonia* (10.8%), *Leptothrix* (5.6%), *Sphingomonas* (4.3%), *Methylotenera* (3.7%), *Enterobacter* (3.2%), *Escherichia*-*Shigella* (3.2%) and uncultured bacteria (3.0%). The relative abundances of the genera *Methylobacterium* (41.1%), *Enterobacter* (10.6%) and *Sphingomonas* (5.9%) in the AEFM group were higher than those in the AEFW group. Among the *Ae. albopictus* groups, the bacterial composition of ALMW was dominated by the genera *Wolbachia* (37.7%), *Bacillus* (22.9%), *Enterobacter* (14.8%) and *Methylobacterium* (8.2%), and the ALFW mosquitoes were dominated by *Wolbachia* (78.7%), *Methylobacterium* (7.8%) and *Enterobacter* (4.1%). The ALFM mosquitoes had higher abundances of *Methylobacterium* (67.4%) and *Sphingomonas* (7.9%) and lower abundances of *Wolbachia* (9.5%) and *Enterobacter* (0.2%) than the ALFW mosquitoes. Our results showed that the dominant microbes colonizing the entire bodies of mosquitoes were distinct and associated with the host’s genetic background. The midguts of female *Aedes* mosquitoes harbored similarly dominant microbes, which were not dependent on the species or genetic type.

### Bacterial community structures of *Aedes* mosquitoes

**Fig. 2:**
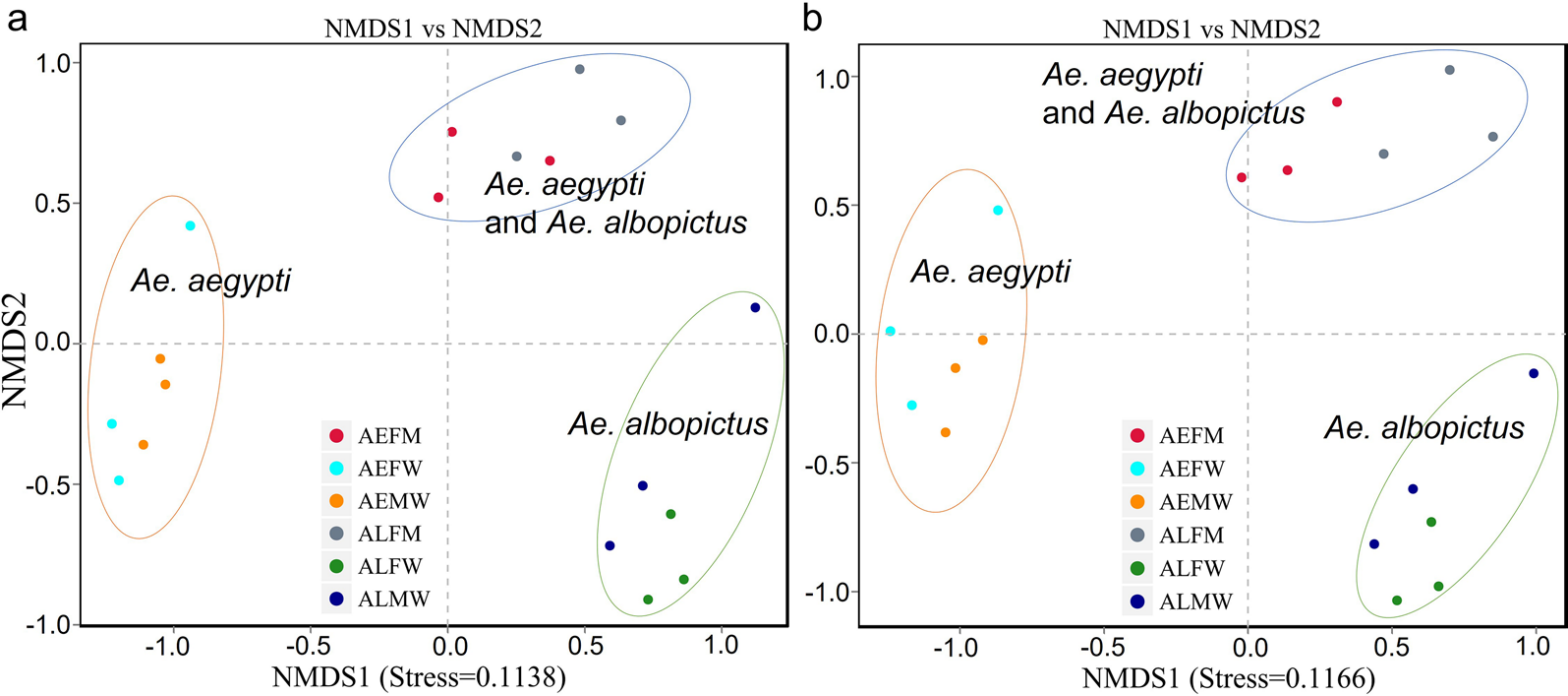
NMDS analysis showing microbiome communities of each sample. **a** Based on the genus level (stress = 0.1138). **b** Based on the OTU level (stress = 0.1166). The blue ellipse indicates that the bacterial structures in the midgut of both *Ae. aegypti* and *Ae. albopictus* are similar to but distinct from those in entire bodies of *Aedes* mosquitoes. The orange and green ellipses indicated that similar bacterial structures were harbored in the entire bodies of both male and female mosquitoes of *Ae. aegypti* and *Ae. albopictus*, respectively. AEMW: entire body of male *Ae. aegypti*. AEFW: entire body of female *Ae. aegypti*. AEFM: midgut of female *Ae. aegypti*. ALMW: entire body of male *Ae. albopictus*. ALFW: entire body of female *Ae. albopictus*. ALFM: midgut of female *Ae. albopictus*

To investigate the bacterial community structures among samples, we performed NMDS analysis (quantified by Bray-Curtis dissimilarity). The NMDS results (Fig. 2) showed that the bacterial communities in the entire bodies of *Ae. aegypti* and *Ae. albopictus* were significantly different, while the microbiota compositions of the entire bodies of both the male and female *Ae. aegypti* and *Ae. albopictus* were similar. These findings demonstrated that the similarity of the bacterial community structure in the entire body of the *Aedes* mosquito was associated with the species and genetic type. Interestingly, we found that the bacterial communities in the midguts of *Ae. aegypti* and *Ae. albopictus* were highly similar and the bacterial structures in the midgut were significantly different from those in the entire body (Fig. 2). These results showed that the composition and structure of the midgut of *Aedes* were not dependent on the species or genetic type.

**Fig. 3:**
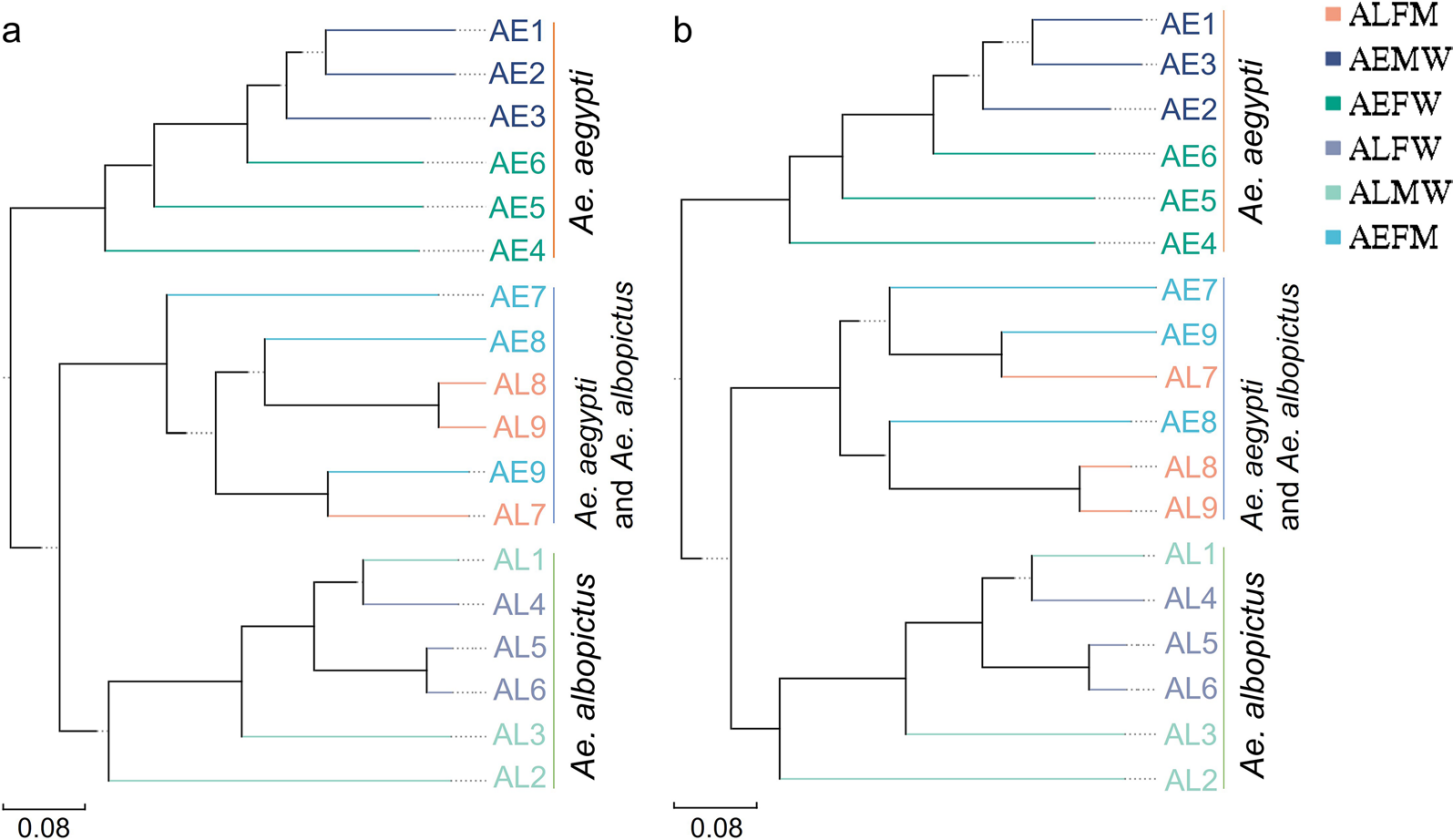
UPGMA analysis based on unifrac distance showing the cluster of microbiome communities in *Aedes* samples. **a** Based on the genus. **b** Based on the OTUs. The blue standing string indicates that the bacterial structures in the midgut of both *Ae. aegypti* and *Ae. albopictus* are similar and convergent but distinct in relation to that in entire bodies of *Aedes* mosquitoes. The orange and green standing strings indicate that similar bacterial structures are harbored in the entire bodies of both male and female mosquitoes of *Ae. aegypti* and *Ae. albopictus*, respectively. AEMW: entire body of male *Ae. aegypti*. AEFW: entire body of female *Ae. aegypti*. AEFM: midgut of female *Ae. aegypti*. ALMW: entire body of male *Ae. albopictus*. ALFW: entire body of female *Ae. albopictus*. ALFM: midgut of female *Ae. albopictus*. AE: *Ae. aegypti*. AL: *Ae. albopictus*

To authenticate the above results, we performed UPGMA analysis. Our findings (Fig. 3) showed that the composition structures in the entire bodies of both males and females of the same *Aedes* species were clustered in the same branches, while the bacterial communities in the entire bodies of *Ae. aegypti* and *Ae. albopictus* were divided into different clusters. Interestingly, the bacterial structures of different samples in the midgut of *Aedes* mosquitoes were under the same clusters, showing a convergent gut microbiota in *Aedes* mosquitoes (Fig. 3). These results were similar to the findings shown by NMDS analysis. Our work found a higher similarity of microbiota composition harbored in the midgut compared with that in the entire body of the *Aedes* mosquito. Our study suggested that the microbiota shaping by the environment in tissue was more than that in the entire body of the *Aedes* mosquito.

### Microbial diversity of *Aedes* mosquitoes

**Fig. 4:**
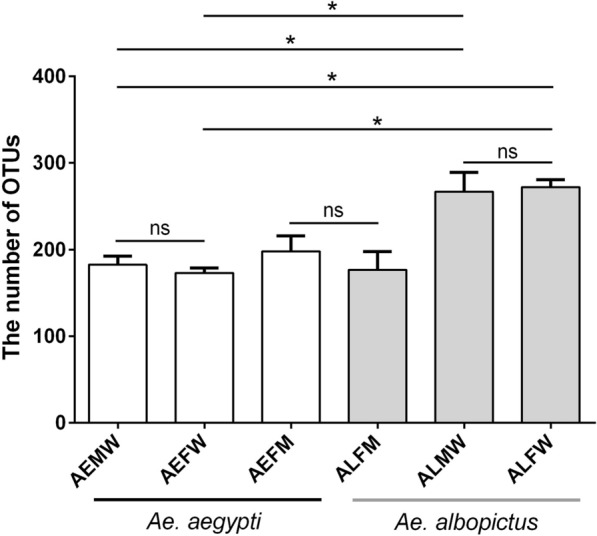
Number of OTUs is shown in each group. * Significant difference determined by the Student’s t-test at *P*-value < 0.05. AEMW: entire body of male *Ae. aegypti*. AEFW: entire body of female *Ae. aegypti*. AEFM: midgut of female *Ae. aegypti*. ALMW: entire body of male *Ae. albopictus*. ALFW: entire body of female *Ae. albopictus*. ALFM: midgut of female *Ae. albopictus*

To examine the alpha diversity between *Ae. aegypti* and *Ae. albopictus*, we rarified the OTU table and calculated the richness using two metrics: ACE and Shannon index. Good’s coverage was > 99.98% for each sample. No significant difference was found in the bacterial alpha diversity in the entire body of either the male or female *Ae. aegypti* or *Ae. albopictus*. However, the diversities of the bacterial communities in the entire bodies of *Ae. albopictus* groups (including ALFW and ALMW) were higher than those of *Ae. aegypti* groups (including AEFW and AEMW) (Table 1). Our findings indicated that there was a significant difference in bacterial diversity between *Ae. aegypti* and *Ae. albopictus*, showing that *Ae. albopictus* harbored more bacterial richness than *Ae. aegypti*. The diversity of the midgut microbiota was higher than the diversities in the entire bodies of both male and female *Ae. aegypti* (Table 1). Interestingly, there were significant differences in bacterial diversities between the entire body and midgut of female *Ae. albopictus*, between the entire body of male *Ae. albopictus* and midgut of female *Ae. albopictus* and between the entire body of female *Ae. albopictus* and midgut of female *Ae. aegypti*, while no significant difference was found in the midgut microbiota between *Ae. aegypti* and *Ae. albopictus* (Table 1). These results demonstrated that higher bacterial diversity existed in the entire body than in the midgut of *Aedes*, showing a similar bacterial diversity in the midgut. We found similar results on the number of OTUs of microbiomes in *Aedes* mosquitoes (Fig. 4). Overall, our findings showed that the midgut microbiota was shaped by environmental factors to a greater extent than the entire body of *Aedes* mosquitoes.

**Table 1:** Diversity indices and Good’s coverage of the bacterial composition of *Ae. albopictus* and *Ae. aegypti*

Group	ACE (Mean ± SD)	Shannon (Mean ± SD)	Coverage (%)
AEMW	182.6 ± 5.6	4.42 ± 0.18	99.98
AEFW	173.1 ± 3.3	4.23 ± 0.29	99.98
AEFM	197.9 ± 10.3	3.46 ± 0.28	99.99
ALMW#	266.7 ± 12.9	2.81 ± 0.16	99.98
ALFW*	272.0 ± 5.0	2.39 ± 0.19	99.99
ALFM	176.6 ± 12.2	2.30 ± 0.13	99.99

^#^Significant differences (*P* < 0.05) in microbiota diversities between ALMW and AEMW, between ALMW and AEFW and between ALMW and ALFM

*Significant differences in bacterial diversities between ALFW and AEFW, between ALFW and AEMW and between ALFW and ALFM

**Fig. 5:**
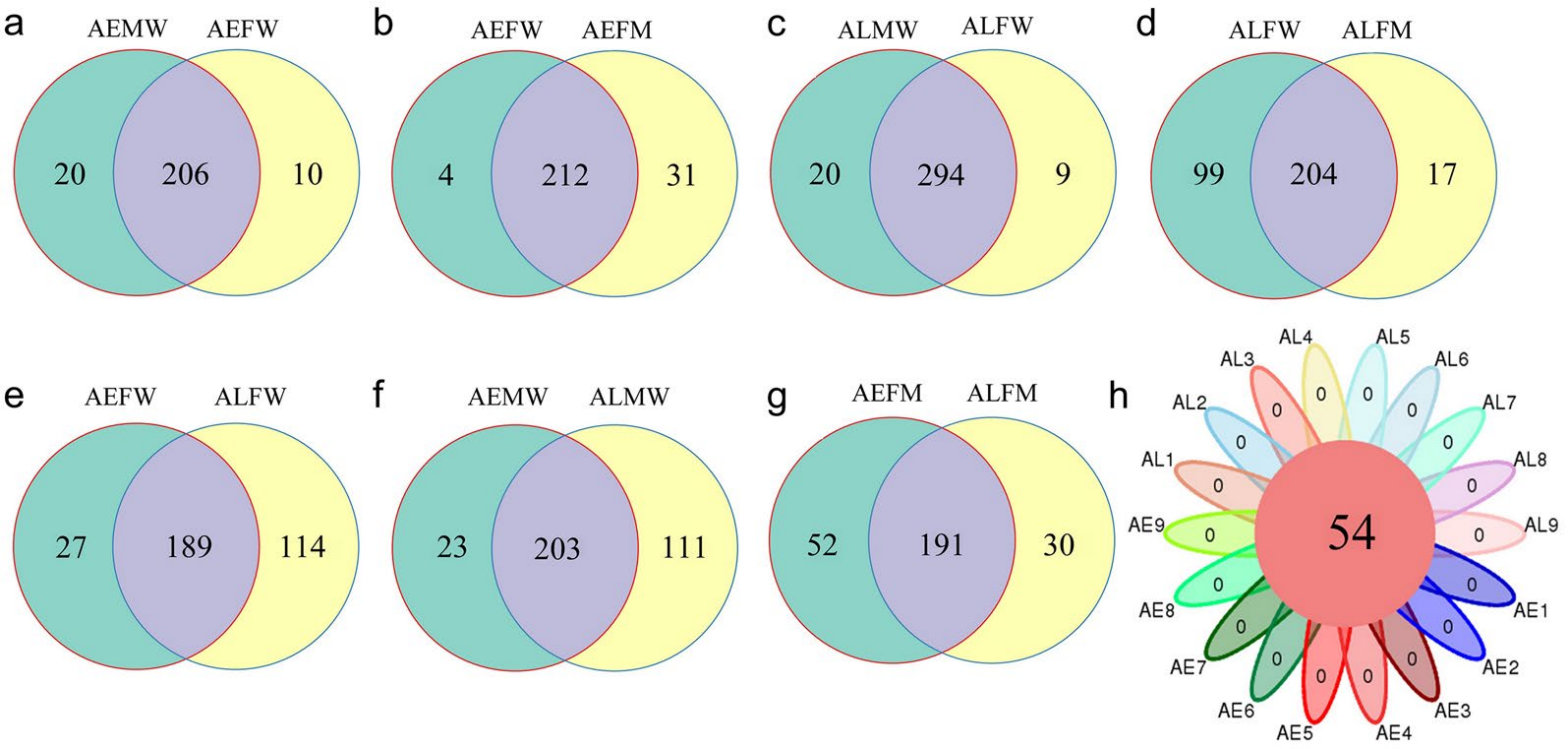
Venn diagrams showing the common and unique OTUs between groups. **a** Between AEMW and AEFW. **b** Between AEFW and AEFM. **c** Between ALMW and ALFW. **d** Between ALFW and ALFM. **e** Between AEFW and ALFW. **f** Between AEMW and ALMW. **g** Between AEFM and ALFM. **h** Venn diagrams showing the core OTUs among samples. AEMW: entire body of male *Ae. aegypti*. AEFW: entire body of female *Ae. aegypti*. AEFM: midgut of female *Ae. aegypti*. ALMW: entire body of male *Ae. albopictus*. ALFW: entire body of female *Ae. albopictus*. ALFM: midgut of female *Ae. albopictus*. AE: *Ae. aegypti*. AL: *Ae. albopictus*

In addition, we found that most OTUs were shared in the entire bodies of male and female *Aedes* mosquitoes, and similar results were observed in the entire body and midgut of *Aedes* mosquitoes (Fig. 5a–d). However, more unique OTUs existed in the midgut than in the entire body of *Ae. aegypti* (Fig. 5b). There were more unique OTUs existing in the entire body than in the midgut of *Ae. albopictus* (Fig. 5d). In addition, the number of unique OTUs harbored by females and males of *Ae. albopictus* was larger than that of *Ae. aegypti*, with counts of 114 and 111, respectively (Fig. 5e, f). This finding showed that the number of unique OTUs between different species was larger than that between the same species (Fig. 5a, c, e, f). Interestingly, the numbers of unique OTUs in the midgut microbiota of *Ae. albopictus* and *Ae. aegypti* were similar (Fig. 5g) and were smaller than those in the entire bodies of *Aedes* mosquitoes (Fig. 5e–g). Finally, only 54 OTUs were shared among all samples (Fig. 5h). These findings demonstrated that there was a high similarity in the bacterial composition of the entire body and the midgut of mosquitoes in the same species, regardless of sex. The difference in the compositions of the midgut microbiota between female *Ae. albopictus* and *Ae. aegypti* was smaller than that in the entire body.

### Functional gene prediction of bacterial composition in *Aedes* mosquitoes

**Fig. 6:**
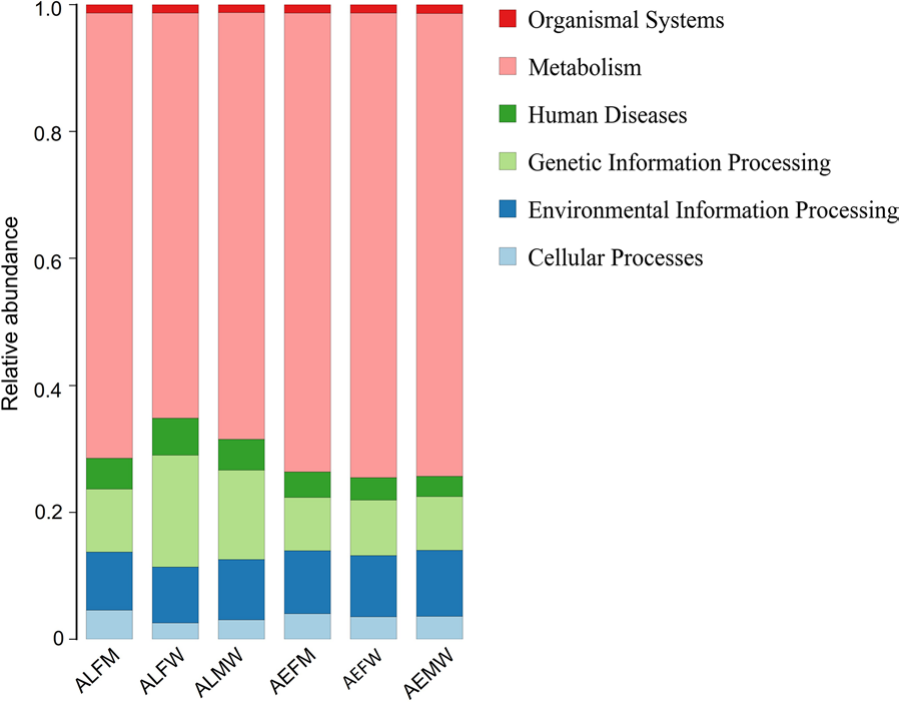
Level 1 of predicted function categories is shown in each group. AEMW: entire body of male *Ae. aegypti*. AEFW: entire body of female *Ae. aegypti*. AEFM: midgut of female *Ae. aegypti*. ALMW: entire body of male *Ae. albopictus*. ALFW: entire body of female *Ae. albopictus*. ALFM: midgut of female *Ae. Albopictus*

The functional genes of bacterial communities of the *Aedes* mosquitoes were explored based on OTUs using PICRUSt software [33, 34]. Functional categories classified into level 1 functional categories were enriched in metabolism, environmental information processing, genetic information processing, cellular processes, human diseases, and organismal systems (Fig. 6). The predicted functions of all groups (AEMW, AEFW, AEFM, ALMW, ALFW and ALFM) were mainly identified in metabolism (72.92%, 73.16%, 72.31%, 66.79%, 63.85% and 70.28%, respectively), the second most common was environmental information processing (13.88%, 13.35%, 13.21%, 16.16%, 17.20% and 13.21%, respectively), and the third most common was genetic information processing (4.96%, 5.10%, 5.15%, 7.86%, 9.26% and 5.77%, respectively).

**Fig. 7:**
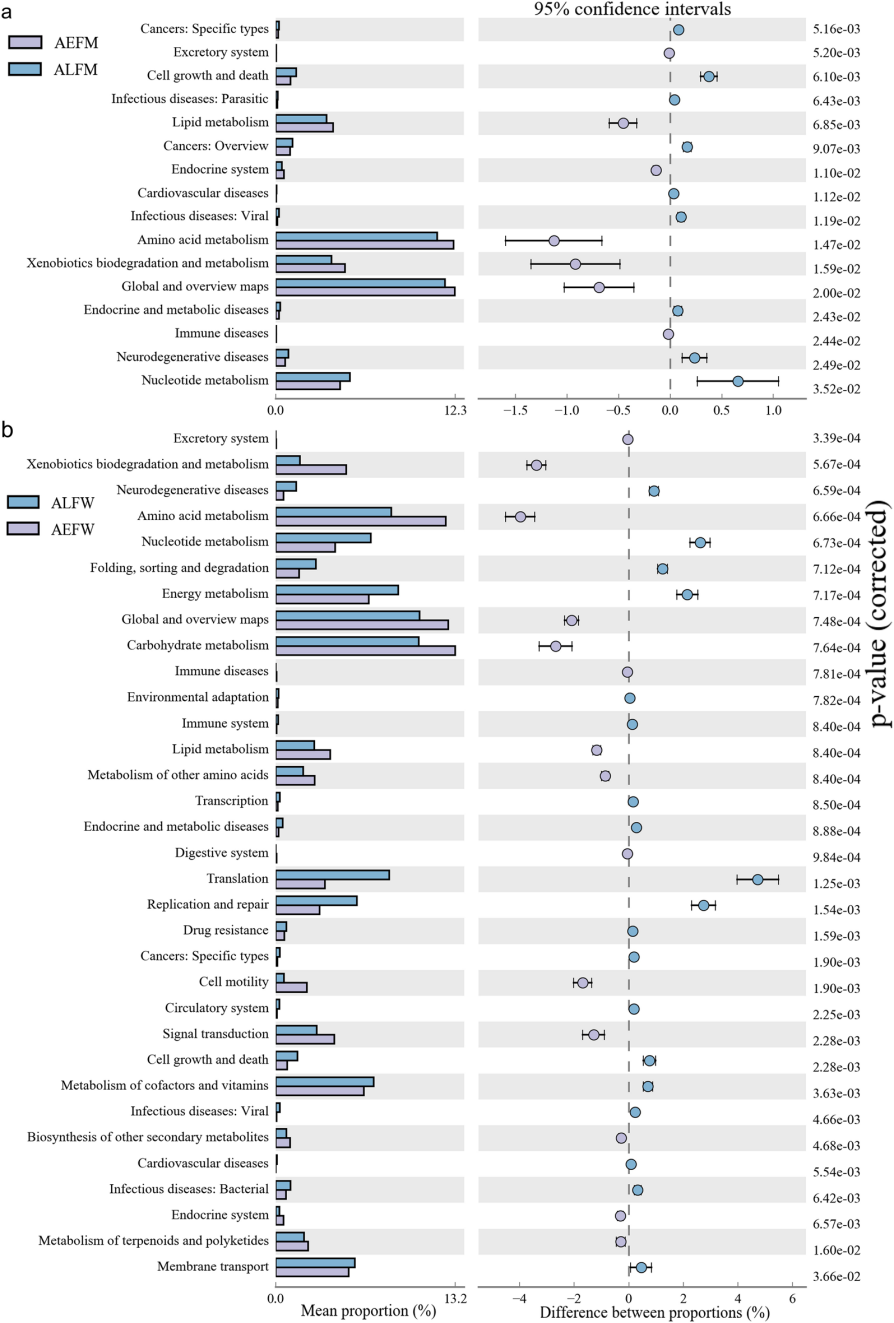
Significantly different distribution of level 2 of predicted functional categories between groups (*P* < 0.05). **a** Sixteen items of functional category between AEFM and ALFM. **b** Thirty-three items of functional category between AEFW and ALFW. AEFM: midgut of female *Ae. aegypti*. ALFM: midgut of female *Ae. Albopictus*. AEFW: entire body of female *Ae. aegypti*. ALFW: entire body of female *Ae. albopictus*

Moreover, the pathways of carbohydrate metabolism, global and overview maps, amino acid metabolism, energy metabolism and metabolism of cofactors and vitamins were among the most abundant functional classes belonging to level 2 (Fig. 7). To further detect the significantly different abundances of metabolic pathways between groups, we used a t-test to compare the functional pathways. We found there were 16 significantly different pathways of Kyoto Encyclopedia of Genes and Genomes (KEGG) categories in the midgut microbiota between *Ae. albopictus* and *Ae. aegypti* (Fig. 7a). The abundances of the level 2 KEGG pathways, such as amino acid metabolism, xenobiotic biodegradation and metabolism, global and overview maps, endocrine and metabolic diseases, immune diseases, neurodegenerative diseases and nucleotide metabolism, were significantly different. There were 33 significantly different KEGG pathways of microbiota composition in the entire bodies of female *Ae. albopictus* and *Ae. aegypti* (Fig. 7b). Significantly different pathways, such as excretory system, xenobiotic biodegradation and metabolism, neurodegenerative diseases, amino acid metabolism and nucleotide metabolism, were noted. In addition, there were 24, 29 and 248 significantly different level 3 KEGG pathways in the comparisons between ALFM and AEFM (Additional file 3: Figure S2), between ALMW and AEMW (Additional file 4: Figure S3) and between ALFW and AEFW (Additional file 5: Figure S4), respectively. Our findings showed that the difference in predicted functions of midgut microbiota was smaller than that of the entire body microbiota of *Aedes* mosquitoes under the same rearing conditions.

### Co-occurrence between the genus *Wolbachia* and other microbes of *Ae. albopictus*

**Fig. 8:**
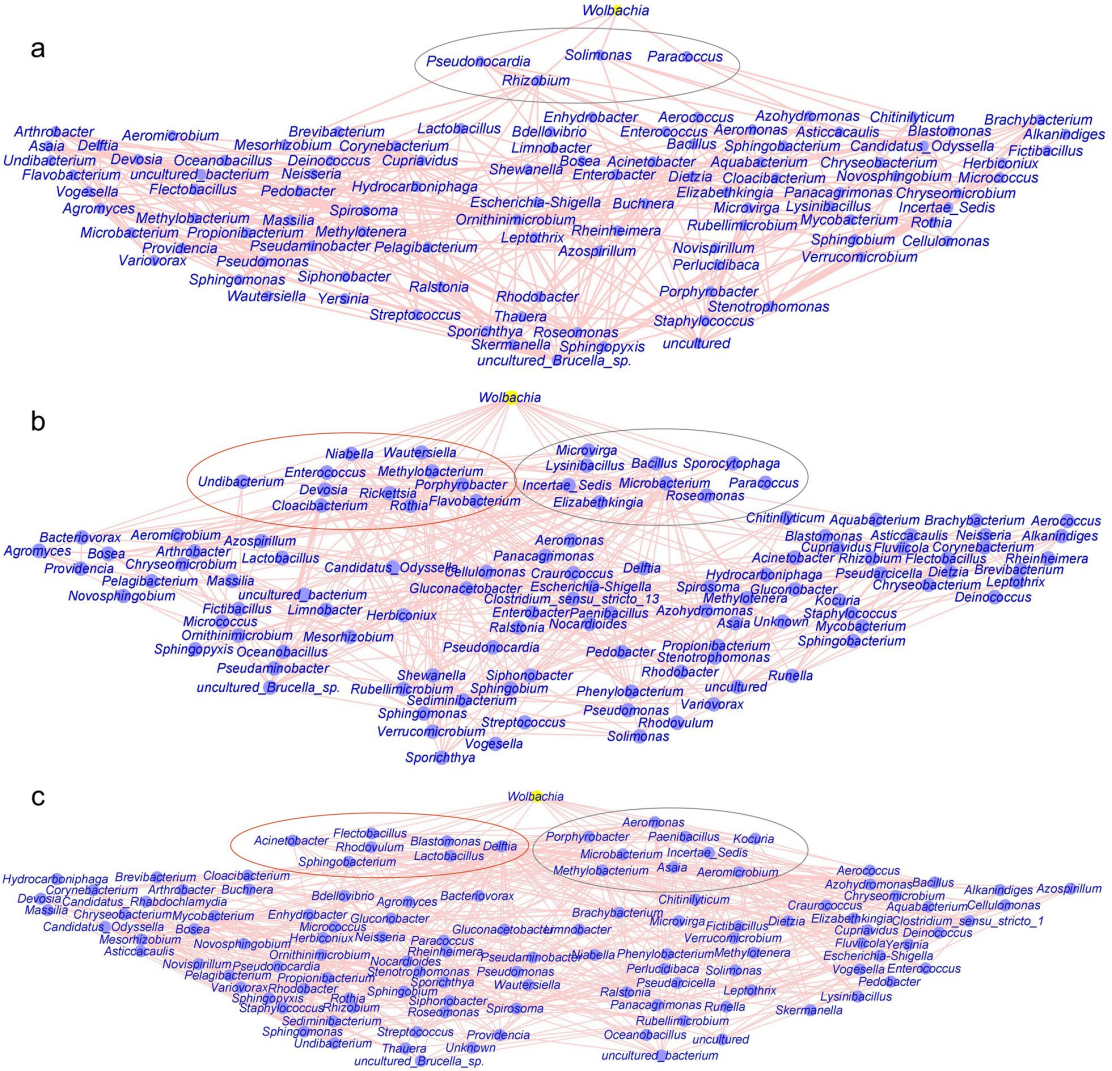
Network analysis between genus *Wolbachia* and bacterial communities of *Ae. albopictus*. **a** In the midgut of female mosquitoes containing 101 nodes and 379 edges. **b** In the entire female mosquitoes containing 111 nodes and 458 edges. **c** In the entire male mosquitoes containing 115 nodes and 607 edges. All edges are statistically significant (*P* < 0.05). The red circle represents these microbiota, which were positively associated with the genus *Wolbachia*. The gray circle represents a negative correlation between the genus *Wolbachia* and the microbes

The genera *Bacillus*, *Methylobacterium* and *Wolbachia* were dominant microbes in *Ae. albopictus* (Additional file 6: Figure S5). The bacterium *Wolbachia* plays an important role in blocking the transmission of Zika and dengue virus. Therefore, we built a network analysis to further investigate the co-occurrence networks of the genus *Wolbachia* and bacterial communities in *Ae. albopictus* (Fig. 8). The results showed that the bacterial community of the entire body of male *Ae. albopictus* had more edges than that of female *Ae. albopictus*. The number of edges observed in the entire body of microbiota of mosquito groups was greater than that in the midgut of *Ae. albopictus*. We found that the genus *Wolbachia* was negatively correlated with *Solimonas*, *Rhizobium*, *Pseudonocardia* and *Paracoccus* (Fig. 8a). Network analysis of the microbiota community of the entire body of female *Ae. albopictus* showed that the microbe *Wolbachia* was positively associated with *Wautersiella*, *Undibacterium*, *Rothia*, *Rickettsia*, *Porphyrobacter*, *Niabella*, *Methylobacterium*, *Flavobacterium*, *Enterococcus*, *Devosia* and *Cloacibacterium* and negatively associated with *Sporocytophaga*, *Roseomonas*, *Paracoccus*, *Microvirga*, *Microbacterium*, *Lysinibacillus*, *Incertae Sedis*, *Elizabethkingia* and *Bacillus* (Fig. 8b). In addition, the associations of the bacterial community in the male *Ae. albopictus* between *Wolbachia* and *Sphingobacterium*, *Rhodovulum*, *Lactobacillus*, *Flectobacillus*, *Delftia*, *Blastomonas* and *Acinetobacter* were positive, while *Wolbachia* was negatively associated with *Porphyrobacter*, *Paenibacillus*, *Microbacterium*, *Methylobacterium*, *Kocuria*, *Incertae Sedis*, *Asaia*, *Aeromonas* and *Aeromicrobium* (Fig. 8c). Overall, there was strong and complex connectivity between *Wolbachia* and other microbes, indicating competing relationships in the bacterial communities of *Ae. albopictus*.

## Discussion

*Aedes aegypti* and *Ae. albopictus* are among the most unneglected vectors of arboviral diseases and are considered primary public health issues [2, 35]. In addition, *Ae. aegypti*, as an invasive species, has already spread in Guangdong, Yunnan and Hainan provinces in recent years [36] and may transmit pathogens causing microcephaly and other public health problems [37–39]. As the dominant species of mosquitoes in South China, *Ae. albopictus* caused outbreaks of dengue fever in tens of thousands of patients in Guangdong Province in 2014 [40–42]. The gut microbiota, which plays an important role in interrupting disease transmission, affects the interaction between the host and pathogens, such as ZIKV and *Plasmodium* [5, 15–17]. Therefore, it is necessary to investigate the diversity and composition of the microbiota in *Aedes* species in South China. In the present study, we aimed to detect and compare the bacterial communities and diversity in both the entire body and midgut of *Aedes* mosquitoes using the V3-V4 variable region of 16S rRNA gene sequencing.

Previous studies showed that the microbiota of mosquitoes were significantly affected by the geographical location [20, 43]. Mosquitoes collected from different geographical regions and habitats harbored significantly diverse microbiota [26, 43, 44]. These previous results indicated that the mosquitoes collected from Hainan and Guangdong provinces may harbor a diverse and distinct composition and diversity of microbiota. The mosquitoes were reared under laboratory conditions for > 30 generations. However, how the midgut and entire body microbiota change when shaped by the laboratory environment is unclear.

We found a lower bacterial diversity of the entire body of *Ae. aegypti* mosquitoes compared to that of *Ae. albopictus*. The dominant phyla in both *Ae. aegypti* and *Ae. albopictus* were *Proteobacteria* and *Bacteroidetes*, the results of which were similar to previous research [45]. The results of similarly dominant phyla of both the entire body and the midgut in *Aedes* mosquitoes indicated that the main microbiota did not depend on the organ, sex or species.

We cataloged the genus microbiota distribution in each group. Common microbes in the entire bodies of both male and female *Ae. aegypti* mainly included seven genera: *Leptothrix*, *Methylobacterium*, *Enterobacter*, *Methylotenera*, *Uncultured bacteria*, *Escherichia Shigella* and *Sphingomonas*. The relative richness of these microbes was > 1%. Meanwhile, the relative abundance of all microbes in *Ae. aegypti* was < 15%, suggesting that the bacterial composition of *Ae. aegypti* was rather decentralized, and no microbes were absolutely dominant. In addition, the genera *Wolbachia* (37.72% and 78.67%, respectively), *Methylobacterium* and *Enterobacter* were the common microbes in the entire bodies of both male and female *Ae. albopictus*, showing higher relative abundances than the genera in *Ae. aegypti*. These results revealed that the bacterial composition of *Ae. albopictus* is concentrated in a few genera, such as *Wolbachia*. Interestingly, the genus *Bacillus* (phylum: *Firmicutes*) was the second most dominant bacteria in male *Ae. albopictus*, while the female mosquito harbored only a few, and this bacterium existed in other species, as reported in previous studies [12, 45].

A previous study revealed that environmental factors dominated host genetics in shaping the host gut microbiota [46]. Recent studies have suggested that a diversity of bacteria-colonizing mosquitoes is acquired from aquatic larval habitats [23, 43]. The environment is an essential factor in researching the bacterial diversity and composition of the host. In addition, the surface of the insect is in contact with the environment and harbors a diverse microbiota [22], and the entire body also houses many microbes [22]. However, the relationship and difference between the entire body and the midgut microbiota of the *Aedes* mosquito are still unclear. How the entire body and the midgut microbiota change under the same living conditions was not clearly revealed. We found a similar midgut microbiota of *Ae. aegypti* and *Ae. albopictus* compared with their entire body microbiota. Our findings revealed that the bacterial diversity and community structures in the midgut of *Ae. aegypti* and *Ae. albopictus* were highly similar and significantly different from those in their entire bodies. Our results suggested that the composition and structure of the midgut of *Aedes* were not dependent on the species or genetic type and that the microbiota shaping in tissue by the environment was more than that in the entire body of the *Aedes* mosquito. Environmental factors can be neglected in microorganism studies of vector biology in both the tissue and the entire body.

Characterization of the functional KEGG pathways detected by the PICRUSt tool [33] is a key element in microbiome research. In this study, the functional genes of the microbiomes of *Aedes* were identified. The level 1 KEGG pathways mainly included metabolism, environmental information processing, genetic information processing, cellular processes, human diseases and organismal systems. These predicted functions were usually detected in the bacterial communities of water and sediments [47]. The study revealed that the metabolism of bacterial communities of fishes is involved in metabolic pathways, environmental information processing and genetic information processing [48]. Previous studies have revealed the importance of microbial communities in nutrient cycling [49] and their metabolites for hosts [50–53]. The microbiota also contributes to the nutrition of insects in different ways [13]. The midgut microbiota can produce compounds such as vitamins, amino acids and sterol, which are directly assimilated by the host [13, 54, 55]. Our results revealed that bacterial symbionts in *Aedes* were associated with the second level of metabolism, including carbohydrate metabolism, global and overview maps, amino acid metabolism, energy metabolism and metabolism of cofactors and vitamins. These metabolic pathways are involved in essential elements for hosts, such as energy supply, amino acid production, cofactors and vitamins. Interestingly, the metabolism levels of bacterial communities in the entire bodies of both male *Ae. aegypti* and *Ae. albopictus* were similar, while they were significantly different in the bacterial compositions of female *Ae. aegypti* and *Ae. albopictus* mosquitoes. However, the mechanism of this result needs to be further tested. Our findings revealed that the difference in predicted functions of midgut microbiota was smaller than that of the entire body microbiota of *Aedes* mosquitoes under the same rearing conditions, showing a similar level on metabolism genes of microbiota. In short, our study suggested that the metabolic functions of microbiota shaped by the environment in tissue were greater than those in the entire body of *Aedes* mosquitoes. However, functional gene prediction based on the 16S rRNA gene was mainly applied to investigate the metabolic pathways of environmental microbiota communities [47]. In our work, we aimed to explain the relationship and difference between the midgut and the entire body microbiota at the molecular level. More experiments and techniques are needed to fully characterize the bacterial metabolism of *Aedes* mosquitoes.

Network analysis represents the complexity of ecological relationships among microbial communities [56, 57]. Previous studies have revealed that *Wolbachia* could block the transmission of Zika and dengue virus [5, 58, 59] and decrease the density of mosquitoes, resulting in disease interruption [60–63], implying the important role of *Wolbachia* in *Aedes*. Here, our study also revealed the dominant genus *Wolbachia.* Our data showed that the relative abundance of *Wolbachia* in *Ae. albopictus* was significantly associated with a few microbes. We found that *Wolbachia* was positively or negatively associated with some microorganisms throughout and in the midgut of *Ae. albopictus*. These results may help us to promote the design of vectors and disease control by modulating the richness of *Wolbachia*.

## Conclusions

In this study, we revealed the composition and diversity of microbiota of *Ae. aegypti* and *Ae. albopictus* from South China. Our finding that *Ae. aegypti* and *Ae. albopictus* reared in the same laboratory harbor a similar midgut bacterial microbiome but different entire body microbiota implies that the gut microbiota of adult mosquitoes is environmentally determined regardless of the host genotype, but the entire body microbiota was influenced more by genotype and less by environment. Our study could provide a further understanding of the aspects of the microbiome of *Aedes* mosquitoes.

## Supplementary Information

**Additional file 1: Figure S1.** Map showing the sampling site of *Ae. aegypti* mosquitoes in Hainan province and the sampling site of *Ae. albopictus* in Guangdong province in 2003. Red triangles represent the sampling sites.

**Additional file 2: Table S1.** Samples of *Aedes* colonies reared in laboratory conditions included in this study.

**Additional file 3: Figure S2.** Significantly different distribution of level 3 of predicted functional categories between AEFM and ALFM (*P* < 0.05).

**Additional file 4: Figure S3.** Significantly different distribution of level 3 of predicted functional categories between AEMW and ALFW (*P* < 0.05)

**Additional file 5: Figure S4.** Significantly different distribution of level 3 of predicted functional categories between AEFW and ALFW (*P* < 0.05).

**Additional file 6: Figure S5.** Relative abundance of dominant bacteria of *Ae. albopictus* compared with each group (*P < 0.05, ***P < 0.0001). (A) Phylum *Firmicutes* and genus *Bacillus*. (B) Genus *Methylobacterium.* (C) Genus *Wolbachia*.

## Data Availability

Please contact the author for additional data requests.

## Abbreviations

WHO: World Health Organization
AEMW: Entire mosquito of male *Ae. aegypti*
AEFW: Entire mosquito of female *Ae. aegypti*
AEFM: Midgut of female *Ae. aegypti*
ALMW: Entire mosquito of male *Ae. albopictus*
ALFW: Entire mosquito of female *Ae. albopictus*
ALFM: Midgut of female *Ae. albopictus*
AE: *Ae. aegypti*
AL: *Ae. albopictus*
NMDS: The nonmetric multidimensional scaling
PICRUSt: Phylogenetic investigation of communities by reconstruction of unobserved states
OTUs: Operational taxonomic units
UPGMA: The unweighted pair-group method with arithmetic mean analysis
KEGG: Kyoto Encyclopedia of Genes and Genomes
