# Supplementary figures and images for: Bacterial composition of midgut and entire body of laboratory colonies of Aedes aegypti and Aedes albopictus from Southern China
Source: Parasit Vectors. 2021 Nov 27;14:586. doi: 10.1186/s13071-021-05050-4 (PMC8626967; doi:10.1186/s13071-021-05050-4)

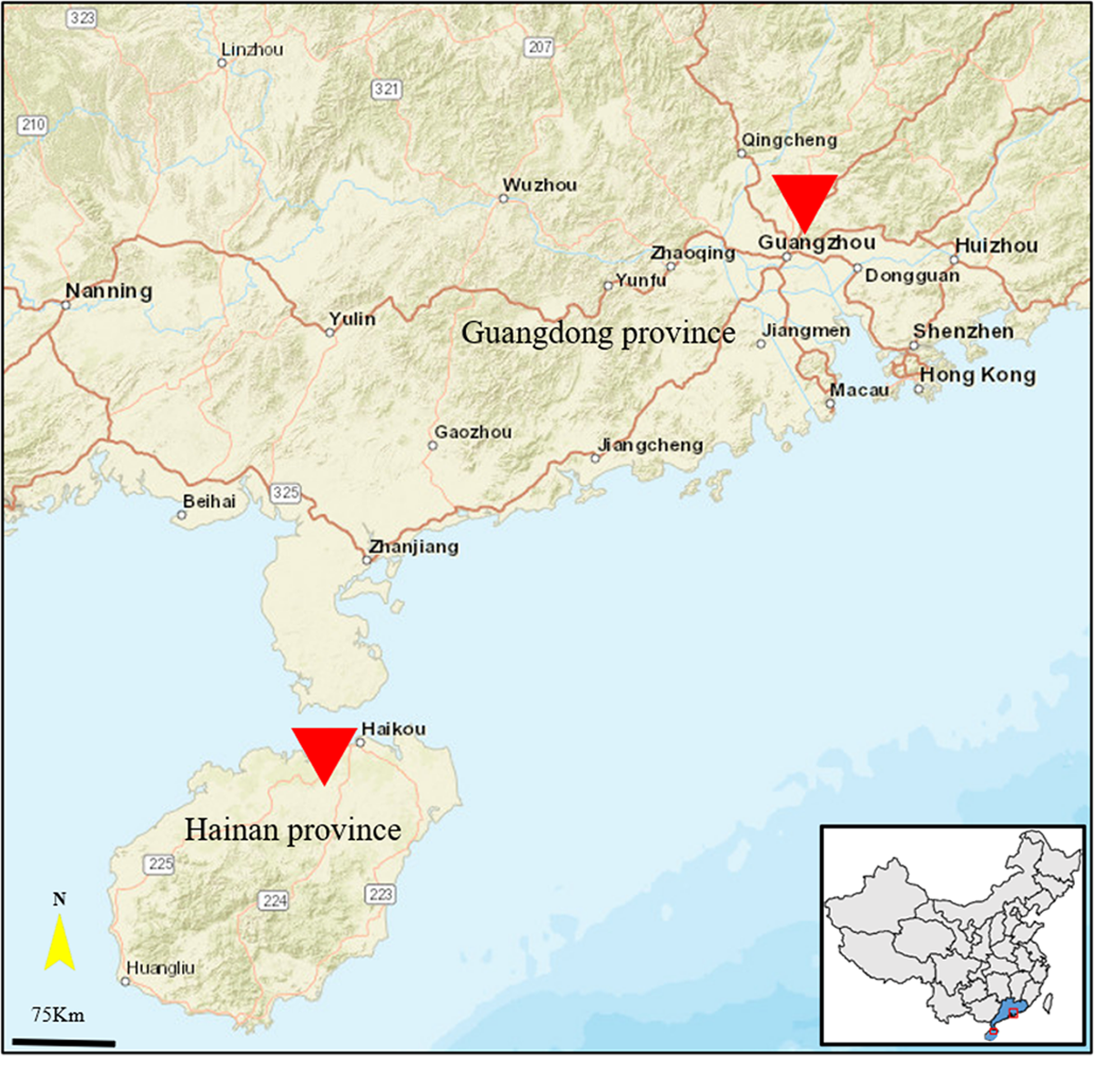

Supplement: Supplementary file 1 — Additional file 1: Figure S1. Map showing the sampling site of Ae. aegypti mosquitoes in Hainan province and the sampling site of Ae. albopictus in Guangdong province in 2003. Red triangles represent the sampling sites. [file 13071_2021_5050_MOESM1_ESM.png]

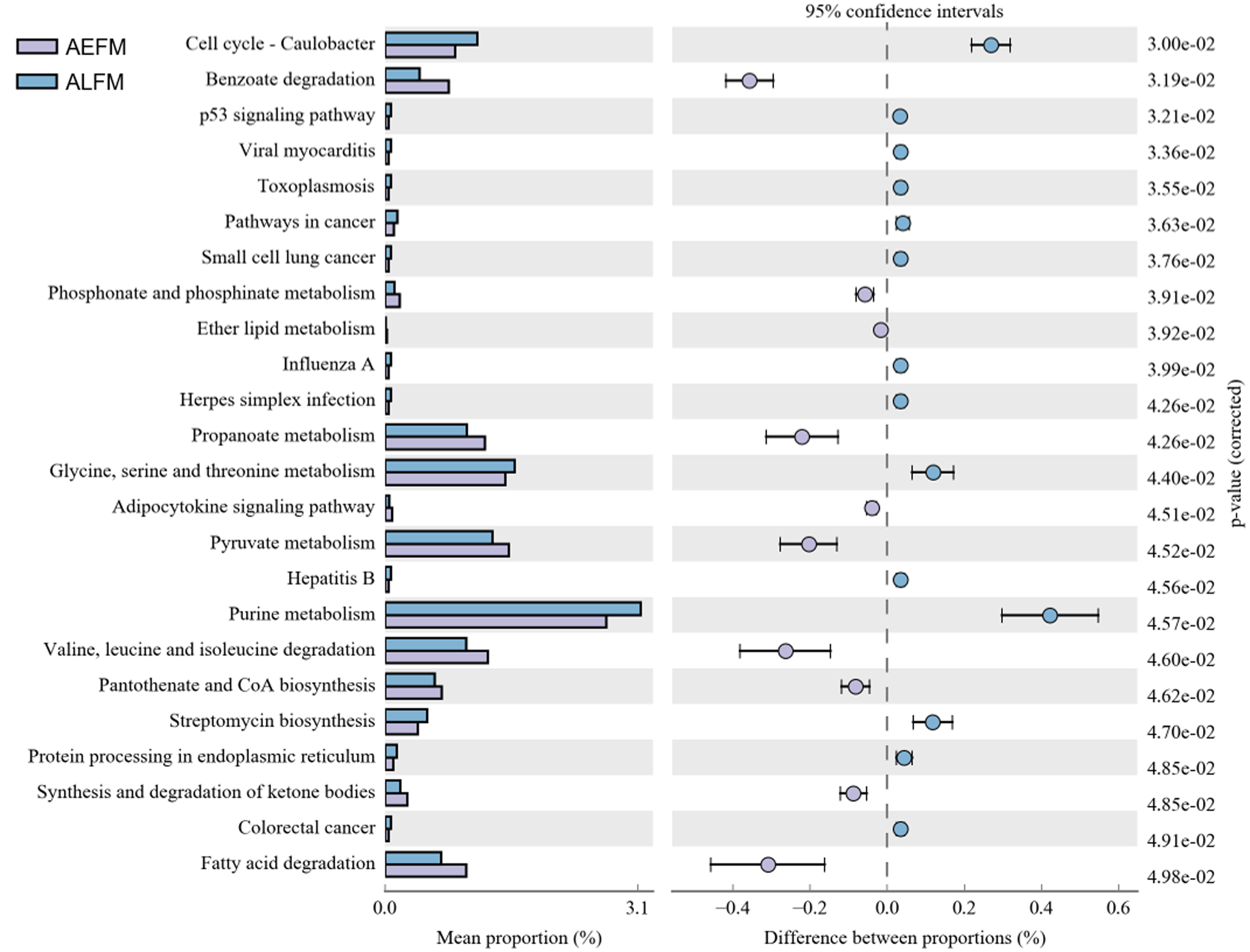

Supplement: Supplementary file 3 — Additional file 3: Figure S2. Significantly different distribution of level 3 of predicted functional categories between AEFM and ALFM (P < 0.05). [file 13071_2021_5050_MOESM3_ESM.png]

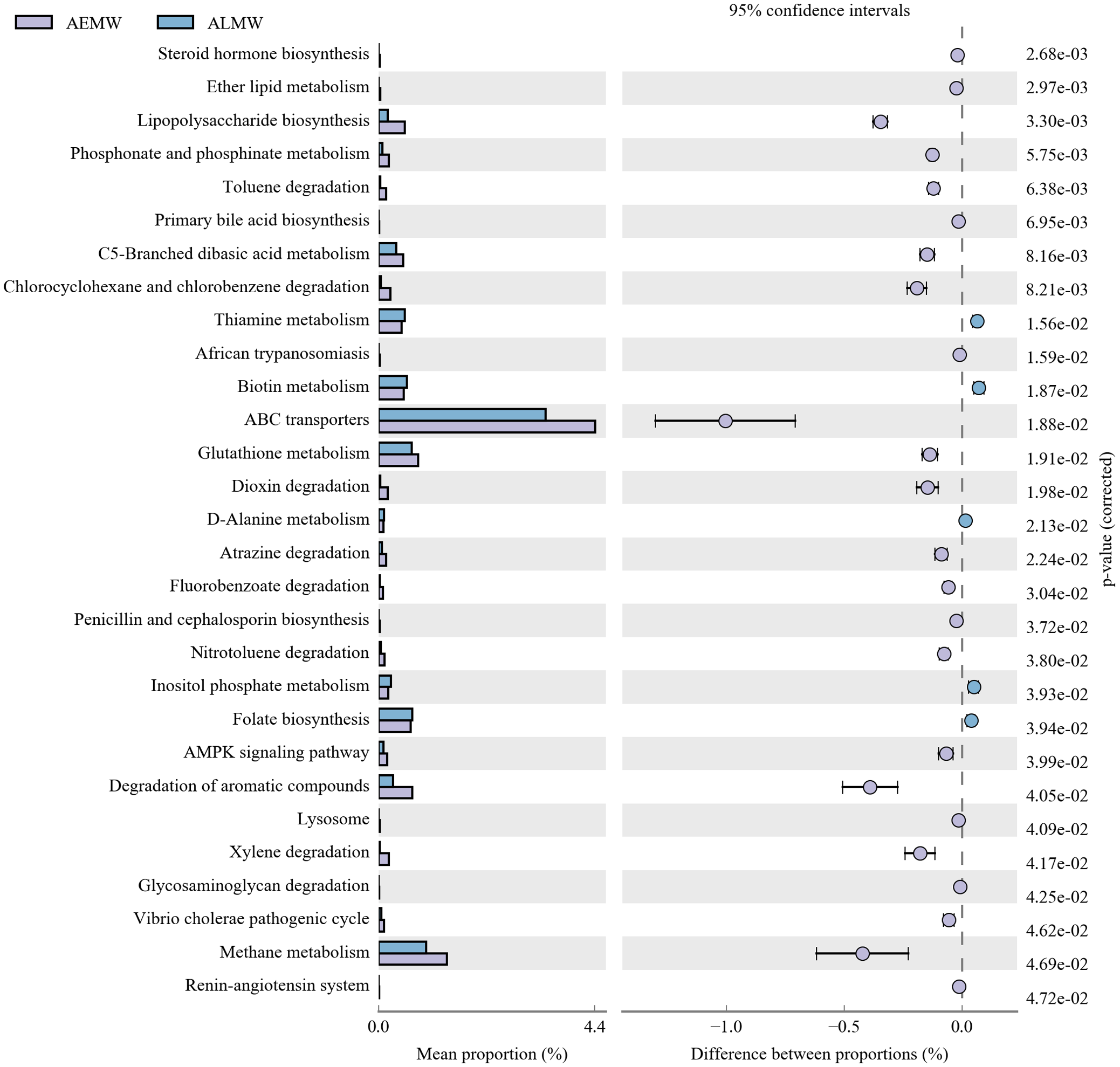

Supplement: Supplementary file 4 — Additional file 4: Figure S3. Significantly different distribution of level 3 of predicted functional categories between AEMW and ALFW (P < 0.05) [file 13071_2021_5050_MOESM4_ESM.png]

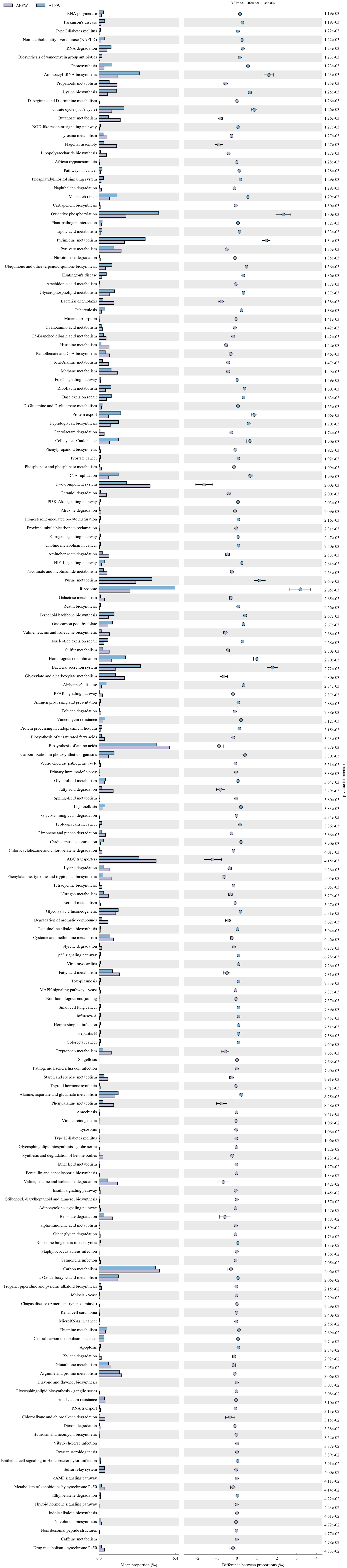

Supplement: Supplementary file 5 — Additional file 5: Figure S4. Significantly different distribution of level 3 of predicted functional categories between AEFW and ALFW (P < 0.05). [file 13071_2021_5050_MOESM5_ESM.png]

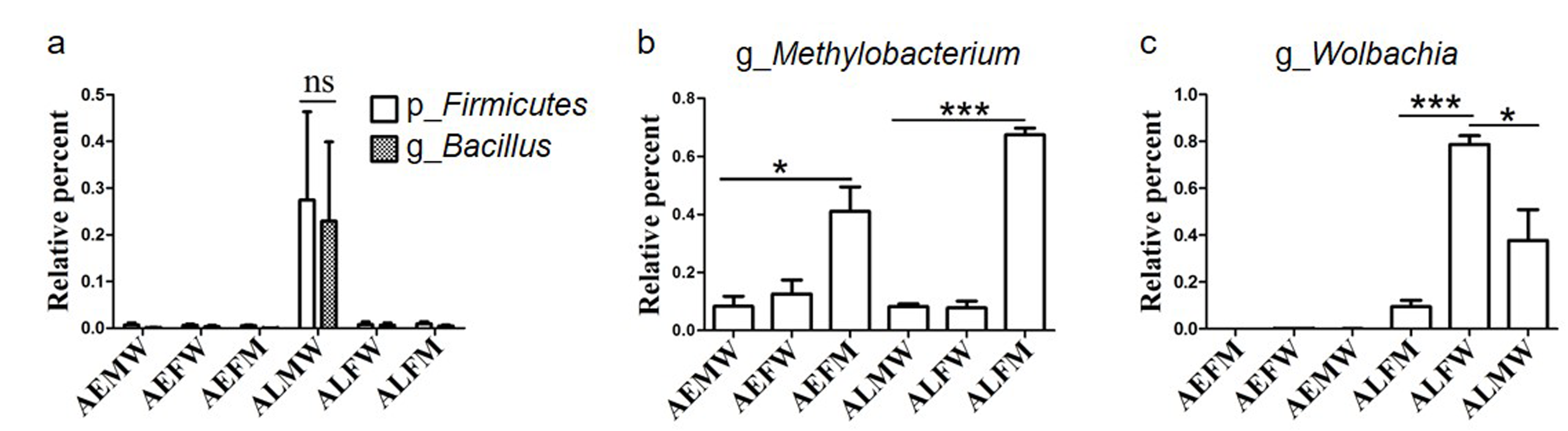

Supplement: Supplementary file 6 — Additional file 6: Figure S5. Relative abundance of dominant bacteria of Ae. albopictus compared with each group (*P < 0.05, ***P < 0.0001). (A) Phylum Firmicutes and genus Bacillus. (B) Genus Methylobacterium. (C) Genus Wolbachia. [file 13071_2021_5050_MOESM6_ESM.png]
