# Supplementary material for: Bacterial composition of midgut and entire body of laboratory colonies of Aedes aegypti and Aedes albopictus from Southern China
Source: Parasit Vectors. 2021 Nov 27;14:586. doi: 10.1186/s13071-021-05050-4 (PMC8626967; doi:10.1186/s13071-021-05050-4)
Supplement: Supplementary file 2 — Additional file 2: Table S1. Samples of Aedes colonies reared in laboratory conditions included in this study. [file 13071_2021_5050_MOESM2_ESM.docx]

**Additional file 2: Table S1.** Sample information of *Aedes* colonies reared under laboratory conditions in this study.

| Sample | Species | Gender | Tissue | Region | Generation |
| --- | --- | --- | --- | --- | --- |
| AEMW | *Aedes aegypti* | Male | Entire mosquito | Hainan province, China | 38 |
| AEFW | *Aedes aegypti* | Female | Entire mosquito | Hainan province, China | 38 |
| ALMW | *Aedes albopictus* | Male | Entire mosquito | Guangzhou, China | 33 |
| ALFW | *Aedes albopictus* | Female | Entire mosquito | Guangzhou, China | 33 |
| AEFM | *Aedes aegypti* | Female | Midgut | Hainan province, China | 38 |
| ALFM | *Aedes albopictus* | Female | Midgut | Guangzhou, China | 33 |

**AEMW:** the entire body of male *Ae. aegypti*. **AEFW:** the entire body of female *Ae. aegypti*. **AEFM**: the midgut of female *Ae. aegypti*. **ALMW:** the entire body of male *Ae. albopictus*. **ALFW:** the entire body of female *Ae. albopictus*. **ALFM:** the midgut of female *Ae. albopictus*.
